# Induction of endothelial barrier dysfunction by serum factors in rats subjected to traumatic brain injury and hemorrhagic shock

**DOI:** 10.14814/phy2.15350

**Published:** 2022-07-03

**Authors:** Yunbo Ke, Julie L. Proctor, Chenou Zhang, Juliana Medina, Catriona H. T. Miller, Junghyun Kim, Thomas E. Grissom, Anna A. Birukova, Gary M. Fiskum, Konstantin G. Birukov

**Affiliations:** ^1^ Department of Anesthesiology University of Maryland School of Medicine Baltimore Maryland USA; ^2^ Division of Pulmonary and Critical Care Medicine Department of Medicine University of Maryland School of Medicine Baltimore Maryland USA

**Keywords:** acute respiratory distress syndrome (ARDS), controlled cortical impact (CCI), endothelial cell barrier dysfunction, hemorrhagic shock (HS), serum, trans‐endothelial resistance (TER), traumatic brain injury (TBI)

## Abstract

Traumatic brain injury (TBI) has been associated with the development of indirect acute respiratory distress syndrome (ARDS). However, the causative relationship between TBI and lung injury remains unclear. To explore potential mechanisms linking TBI with the development of ARDS, we characterized the effects of serum factors released following TBI and hemorrhagic shock (HS) in a rat model on the pulmonary endothelial cell (EC) barrier dysfunction, a key feature of ARDS. We found that serum samples from animals exposed to both controlled cortical impact (CCI) and HS, but not from sham‐operated rats induced significant barrier dysfunction in human pulmonary artery EC monolayers at 2 days post injury. Thrombin inhibitor and thrombin receptor antagonist attenuated the acute phase of the serum‐induced trans‐endothelial resistance (TER) decline caused by CCI‐HS serum, but not in later time points. However, both the early and late phases of CCI‐HS‐induced EC permeability were inhibited by heparin. The barrier disruptive effects of CCI‐HS serum were also prevented by serum preincubation with heparin‐sepharose. Pulmonary EC treated for 3 h with serum from CCI‐HS rats demonstrated a significant decline in expression of EC junctional protein, VE‐Cadherin, and disassembly of peripheral EC adherens junction complexes monitored by immunostaining with VE‐cadherin antibody. These results suggest that exposure to CCI‐HS causes early and late‐phase barrier disruptive effects in vascular endothelium. While thrombin‐PAR1 signaling has been identified as a mechanism of acute EC permeability increase by CCI‐HS serum, the factor(s) defining long‐term EC barrier disruption in CCI‐HS model remains to be determined.

## Graphical abstract

We have identified serum factors from rats subjected to traumatic brain injury (TBI) and hemorrhagic shock caused endothelial barrier dysfunction. Endothelial barrier dysfunction is a key feature of acute respiratory distress syndrome (ARDS). The observation shed light on revealing molecular mechanism that explains TBI‐induced ARDS.

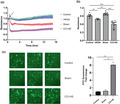

## 1 INTRODUCTION

Traumatic brain injury (TBI) is a leading cause of pervasive morbidity and mortality in both military personnel and civilians that is often characterized by multiple organ dysfunction initiated by brain injury (Meyer et al., 2008; Rutland‐Brown et al., 2006). TBI, especially the severe form as determined by Glasgow outcome scores <8, predisposes to acute respiratory distress syndrome (ARDS), the advanced stage of acute lung injury (Aisiku, Yamal, Doshi, Rubin, et al., 2016). Although it is believed that there must be an intrinsic link between traumatic injury of the brain and the hypoxemic respiratory failure, the definitive molecular events responsible for TBI‐induced ARDS remains unclear (Hu et al., 2017).

ARDS is preceded and accompanied with an increase of lung vascular permeability which contributes to bilateral infusion of fluid between blood and tissues, accumulation of proteins and inflammatory cells in bronchoalveolar lavage (BAL), development of edema and respiratory failure (Maniatis & Orfanos, 2008; Millar et al., 2016). Endotoxin and other exogenous agents trigger cascades of cellular and molecular events leading to typical ARDS phenotypes in mammals (Matute‐Bello et al., 2008). However, emerging evidence has suggested that molecules of internal origin induced under pathological conditions including TBI also play important roles in etiology of ARDS (Fujishima, 2014).

The endothelium forms a continuous monolayer that separates the vascular lumen from the underlying tissues. Normal endothelial cell (EC) barrier function and vascular integrity are essential for the maintenance of efficient gas exchanges of the lungs. The exchange of fluid and cells across the monolayer are tightly regulated under normal physiological conditions. Vascular leak due to breakdown of endothelial barrier integrity leads to interstitial and alveolar edema and compromised lung function as seen in acute lung injury and ARDS (Aman et al., 2016).

EC barrier function is regulated by exogenous and endogenous factors detected in circulation that have profound effects on cytoskeletal reorganization and cell–cell junctions (Stevens et al., 2000). Thrombin is an endogenous bioactive molecule that, besides participation in coagulation cascade increases endothelial permeability by receptor‐mediated mechanisms including activation of actomyosin contraction and cell retractions as well as weakening of adherens junctions (Vandenbroucke et al., 2008). Effects of such edemagenic agonists are counteracted by barrier enhancing signaling molecules including sphingosine 1‐phosphate, high molecular weight hyaluronans, full length oxidized phospholipids, and others (Birukova et al., 2013; Ke et al., 2019).

This study tested the hypothesis that humoral factors appearing in blood circulation of rats subjected to CCI‐HS may induce endothelial barrier dysfunction and trigger TBI‐induced lung barrier dysfunction. The selection of this model of controlled cortical impact (CCI) TBI followed by hemorrhagic shock was informed by several reasons. Most of the severe TBI patients at national shock trauma centers are in shock upon or soon after hospital admission. Despite the substantial incidence of shock following TBI, very few animal models adequately recapitulate this polytrauma. The current study addresses this knowledge gap by utilizing a clinically relevant model of CCI combined with hemorrhagic shock.

We collected fresh blood samples from sham rats and those that underwent CCI in combination with HS. We also analyzed the serum components that induced endothelial barrier dysfunction measured by electric cell‐substrate impedance sensor and cell imaging. Our studies indicate that rats subjected to TBI and HS treatment, but not sham‐operated group, release humoral factors that cause endothelial barrier dysfunction and may be an underlying cause for the development of ARDS in patients with TBI.

## 2 MATERIALS AND METHODS

### 2.1 Reagents and EC culture

Human pulmonary artery endothelial cells (HPAECs) were obtained from Lonza and used in passages 5–8. All experiments were performed in EGM growth medium (Lonza) containing 2% fetal bovine serum unless otherwise specified. Texas Red–conjugated phalloidin and Alexa Fluor 488–labeled secondary antibodies were purchased from Molecular Probes (Eugene). Dabigatran was a gift from MediChemExpress. Thrombin, heparin, heparin‐sepharose, and ML161 were purchased from Sigma. Βeta‐actin monoclonal antibody (Cat no: 66009‐1‐Ig; 1:2000 dilution for WB; 1:100 dilution for IF) was purchased from Proteintech; VCAM‐1(EiE8X) Rabbit monoclonal antibody (Cat no: 12662, 1:1000 dilution for WB) was purchased from Cell Signaling Technology; VE‐Cadherin Polyclonal Antibody (Cat no:160840, 1:500 dilution for WB and IF) was purchased from Cayman Chemical Company.

### 2.2 Animal model and experimental design

The animal model from which the serum samples were collected was designed to be a clinically relevant representation of field TBI in combination with hemorrhagic shock (HS). All animal procedures were performed in accordance with the University of Maryland School of Medicine Institutional Animal Care and Use Committee and approved by the U.S. Air Force Surgeon General's Office of Research Oversight and Compliance regulations. Following induction of deep isoflurane anesthesia in adult male Sprague Dawley rats, a craniotomy was performed, and a catheter was placed in the right femoral artery. Moderate brain injury (2.0 mm depth, 5 m/sec) was induced by controlled cortical impact (CCI). Approximately 2 min later (0 time), blood was removed through the catheter to reach a target MAP of 38–40 mm Hg. HS was maintained within this range for a total of 30 min by removal or addition of small blood volumes (0.5–1.5 ml), when needed. Hypotension was reversed at the onset of the “prehospital” resuscitation phase by infusion of Hextend to maintain MAP at or greater than 55 mm Hg for 1 h. The subsequent 1‐h “hospital” resuscitation phase was initiated by reinfusion of the shed blood, resulting in a further increase in MAP to baseline values. Resuscitation was terminated by cessation of anesthesia. At 24 h after CCI plus hemorrhagic shock, rats were exposed to either normobaria (sea level) or hypobaria (= 8000 ft altitude) for 6 h under normoxic (21–28% O_2_) or hyperoxic (100% O_2_) conditions (Proctor et al., 2015). All sham animals underwent the identical procedures as the animals with the following exceptions. First, while a craniotomy was performed, there was no cortical impact. Second, while small blood samples were taken for blood gas measurements, there was no blood drawn to induce hemorrhagic shock. Shams were exposed to normobaric conditions under 21% O_2_ (Proctor et al., 2015).

### 2.3 Blood and serum collection

Buprenorphine (0.01 mg/kg) was administered postoperatively for analgesia every 8–12 h for 2 days with the first dose administered prior to surgical manipulation. 48 h after the Sham or CCI‐HS procedures, blood was collected from rats heavily anesthetized by intraperitoneal injection of ketamine (160 mg/kg) and xylazine (20 mg/kg) at 2 days postinjury. Blood was drawn from the right ventricles. Rats were euthanized by vital organ perfusion with artificial CSF followed by 4% paraformaldehyde. Blood samples were incubated at room temperature for 30 min followed by centrifugation at 1000*g* for 10 min. Serum samples were aliquoted and stored at −80°C.

### 2.4 Measurement of transendothelial electrical resistance (TER)

Permeability response by pulmonary endothelial cells (EC) to serum treatments was evaluated by monitoring changes in transendothelial electrical resistance (TER) of EC monolayers grown on multi‐well culture plate with golden microelectrodes using the electrical cell‐substrate impedance sensing system (ECIS, Applied Biophysics) as described (Birukova et al., 2007). As cells adhere and spread out on the microelectrode, TER increased (maximal at confluence), whereas cell retraction, rounding, or loss of adhesion was reflected by a decrease in TER. Experiments were conducted only on wells that achieved >1000 Ω (10 microelectrodes per well) of steady‐state resistance. TER values were expressed as normalized resistance as previously described (Birukova et al., 2004). Pulmonary endothelial cells in culture were treated with the sera at 1:50 to 1:200 dilutions in the cell culture medium in the 8‐well array while carrying out TER measurement.

### 2.5 Endothelial permeability assays

Endothelial permeability to macromolecules was monitored by permeability visualization assay (XPerT) available from Millipore (cat. no. 17–10,398). Binding of FITC‐avidin fluorescent tracer to the matrix‐coated culture dish bottoms increases when the EC barrier is compromised. After cell stimulation with serum samples, FITC‐avidin solution at a final concentration of 25 μg/mL was added directly to the culture medium for 3 min before termination of the experiment. Unbound FITC‐avidin was washed out with PBS, pH of 7.4, 37°C (2 cycles, 10 s each). Visualization of FITC‐avidin labeled permeability sites in EC monolayers was performed using inverted microscope Nikon Eclipse TE300 with epifluorescence module and SPOT RT monochrome digital camera and image processor (Diagnostic Instruments). For the 96‐well format assays, the fluorescence of matrix‐bound FITC‐avidin was measured on Victor X5 Multilabel Plate Reader (Perkin Elmer) using an excitation wavelength of 485 nm and emission wavelength of 535 nm (Dubrovskyi et al., 2013).

### 2.6 Immunofluorescence staining

After agonist treatment endothelial cells grown on glass coverslips were fixed in PBS containing 3.7% formaldehyde, and F‐actin was visualized by immunofluorescence staining of cell monolayers with Texas Red conjugated phalloidin. Adherens junctions were labeled with antibodies against VE‐cadherin, as previously described.^**13**^

### 2.7 Western blot analysis

Immunoblot detection of proteins of interest was performed as described previously (Birukova et al., 2007; Ke et al., 2019). In brief, cell lysates of HPAECs cultured with or without serum treatment were subjected to 7.5% SDS‐PAGE, transferred to nitrocellulose membrane in semidry transfer apparatus (Bio‐Rad) 10 V for 1 h, and probed with the antibody of interest. Immunoreactive proteins were visualized by SuperSignal West Dura chemiluminescence reagent according to the manufacturer's protocol (Pierce).

### 2.8 Statistical analysis

Results are expressed as means ± SD of three to eight independent experiments. Stimulated samples were compared with vehicle controls by unpaired Student's *t*‐test. For multiple‐group comparisons, one‐way ANOVA and Tukey's post hoc multiple‐comparison test were used. *p* < 0.05 was considered statistically significant. Because there were no differences in any marker of endothelial barrier dysfunction, serum samples from all four polytrauma groups were pooled into one CCI‐HS group.

## 3 RESULTS

### 3.1 Sera from rats subjected to CCI‐HS treatment induce endothelial barrier dysfunction

**FIGURE 1:**
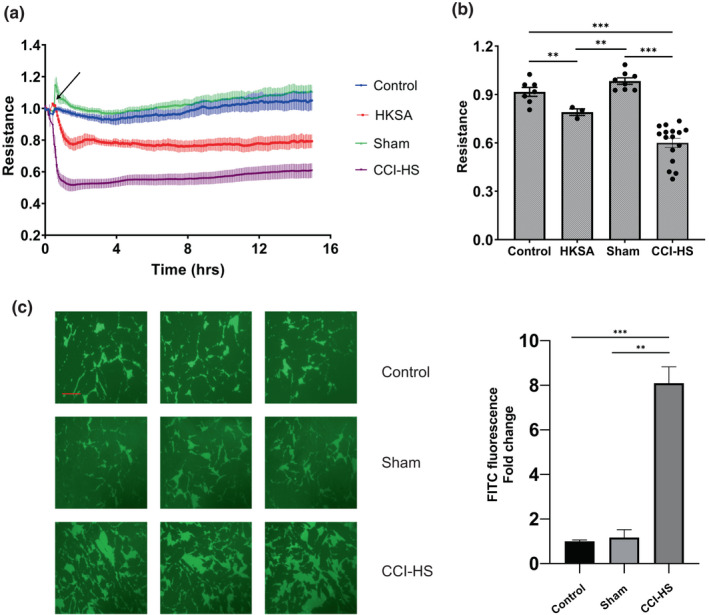
Sera from CCI‐HS rats induce endothelial barrier dysfunction. (a) HPAEC were treated with vehicle, serum samples from CCI‐HS group, sham‐operated group (1:100 final dilution) at the time point marked by arrow or with HKSA as control barrier disruptive agonist. TER was monitored for indicated time periods. Shown are mean ± SEM; *n* = 3. (b) Analysis of TER data at 3 h after the serum stimulation. Dots represent individual TER measurements from independent experiments; **p < 0.01, ***p < 0.001. (c) Effect of CCI‐HS serum on endothelial permeability for macromolecules. Shown on left are representative images from three independent experiments acquired 3 h after serum stimulation. Average fluorescence values and standard errors of three images expressed as fold changes relative to the average value of control are shown on right of the image. **p < 0.01, ***p < 0.001, Bar: 20 μm.

Compared to sham‐operated controls, the rats exposed to CCI‐HS demonstrated acute lung injury 2 days after the trauma characterized by collapsed alveoli, infiltration of red blood cells, and increased inflammatory myeloperoxidase immunoreactivity (Lee & Ansell, 2011). There were no significant changes in serum levels of major cytokines 48 h after the onset of trauma (Proctor et al., 2015; Proctor et al., 2021). The serum samples collected from the CCI‐HS rats and Sham rats were used to test their effects on TER changes in HPAEC. CCI‐HS sera caused a rapid decrease in TER reaching the maximum in less than 1 h after addition of the sera. The TER decline was sustained and lasted at least 15 h after serum challenge. HKSA (heat‐killed *Staphylococcus aureus*) served as a positive control of barrier disruptive agents. Compared to TER reduction caused by HKSA, the CCI‐HS induced TER decrease was more rapid and more pronounced (Figure 1a). Analysis of TER values at 3 hr after serum stimulation showed that TER from the CCI‐HS group was reduced by 34.6% and 39% respectively, as compared to control (non‐operated) and sham‐operated groups (Figure 1b). XPerT permeability visualization assay was further used to evaluate endothelial barrier disruption caused by HPAEC exposure to CCI‐HS serum. HPAEC treatment with serum samples from CCI‐HS, but not from sham‐operated or non‐operated groups induced pronounced disruption of EC monolayer integrity as shown by larger patches of green fluorescence signal (Figure 1C). Dose dependence analysis showed maximal barrier disruptive effect at 1:50–1:200 dilution range. Higher dilution of CCI‐HS sera (1:400–1:800) caused gradual diminishing of the barrier disruptive effect which became undetectable at 1:1600 dilution (data not shown).

### 3.2 PAR1 receptor is involved in acute phase of EC barrier dysfunction caused by CCI‐HS serum

**FIGURE 2:**
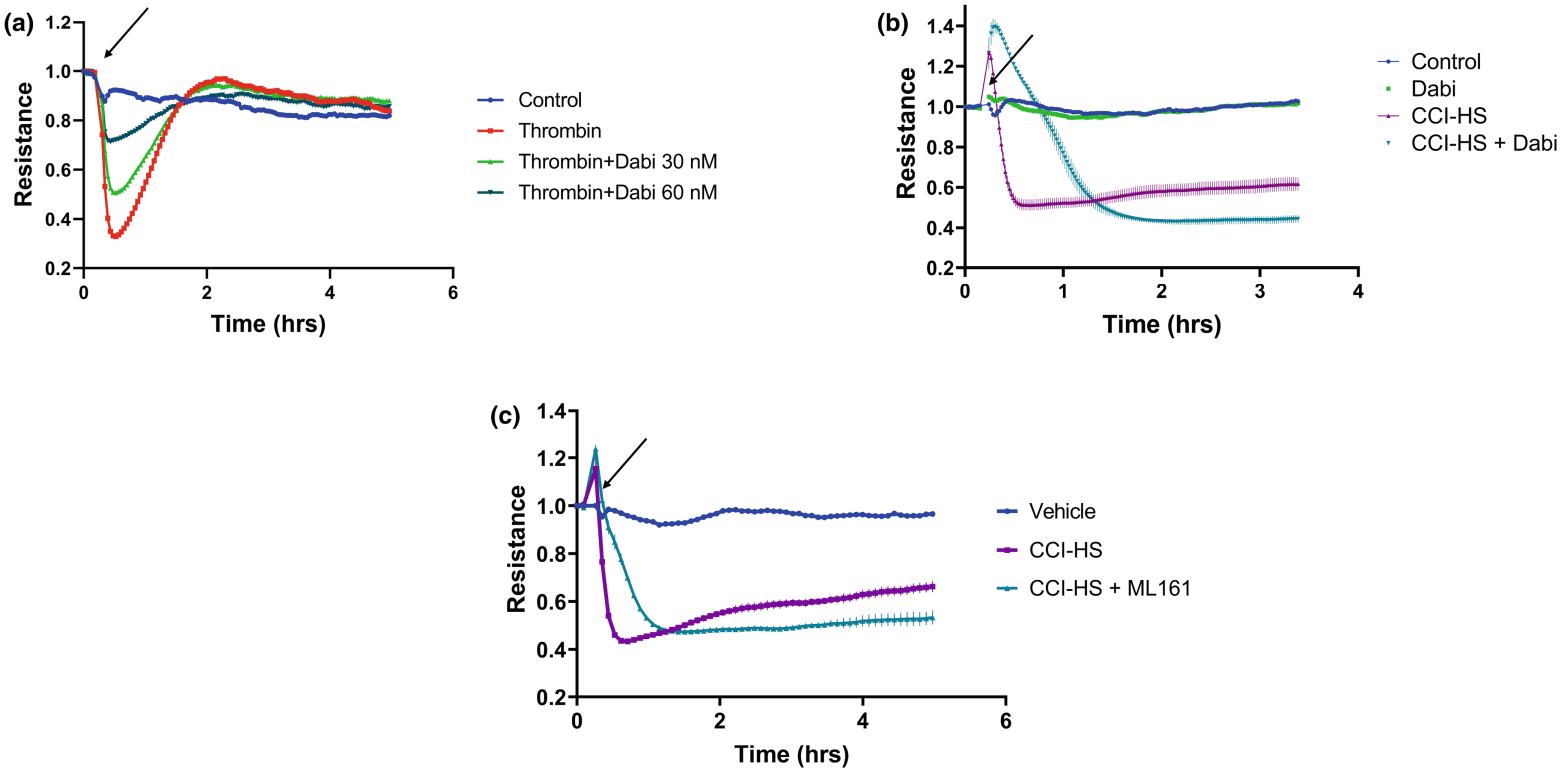
Role of thrombin mechanism in the early phase of HPAEC barrier disruption caused by CCI‐HS sera. CCI‐HS serum (1:100) or thrombin (a) was added to HPAEC with or without pretreatment with: (b) Thrombin inhibitor (dabigatran); or (c) ‐ Par1 antagonist (ML161) at the time point marked by the rrow. TER was monitored for indicated time periods. Shown are representative data from 8 independent measurements.

Because the rapid kinetics of the initial phase of CCI‐HS‐induced HPAEC hyperpermeability resembles the effects of thrombin, and increased thrombin activity often accompanies acute injury syndrome, we investigated the involvement of thrombin mechanism in acute EC barrier dysfunction caused by CCI‐HS. Thrombin‐induced EC barrier dysfunction was attenuated by dabigatran in a dose‐dependent manner (Figure 2a). Dabigatran directly binds to thrombin and inhibits its protease activities (Lee & Ansell, 2011). To test if thrombin is a barrier disruptive component in CCI‐HS serum, HPAEC were treated with CCI‐HS serum alone or together with dabigatran followed by measurement of TER. In the presence of dabigatran, the time to reach maximum decrease in TER by CCI‐HS was delayed by about 1 h (Figure 2b). Accordingly, HPAEC cotreatment with CCI‐HS sera and ML161, an inhibitor for Par1 receptor, caused a similar delay in the peak of TER decline in response to CCI‐HS (Figure 2C). However, both dabigatran and ML161 were unable to affect the late phase of CCI‐HS serum‐induced TER decline beyond 1.5 h post‐treatment (Figure 2b, C).

### 3.3 Heparin attenuates early and late‐phase barrier disruptive activities of CCI‐HS sera

**FIGURE 3:**
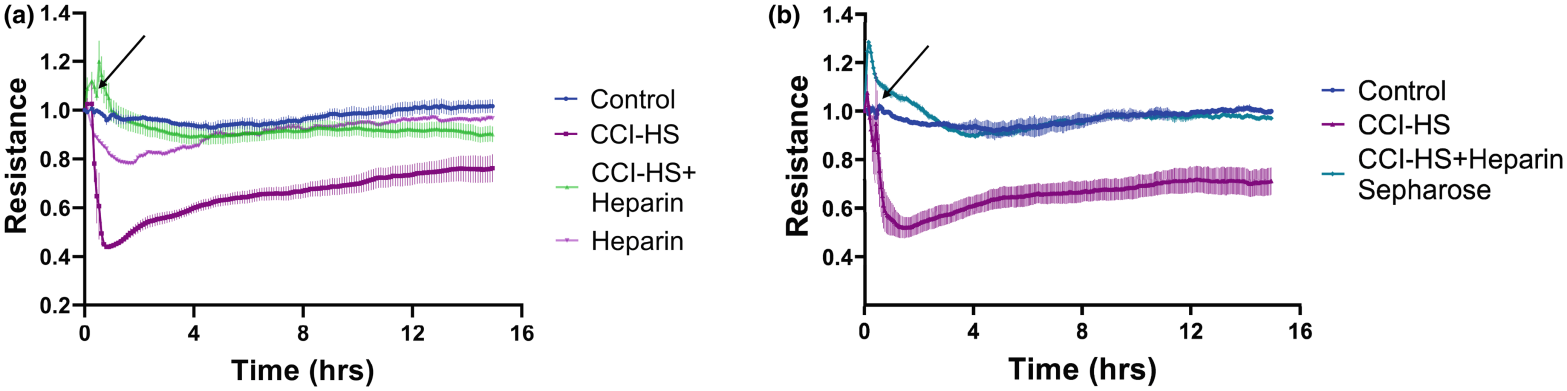
Heparin attenuates CCI‐HS sera‐induced EC barrier dysfunction. (a) HPAEC were stimulated with sera from CCI‐HS rats (1:100) or vehicle with or without co‐treatment with heparin (25 U/mL). TER was monitored for indicated time periods. (b) HPAEC were stimulated with CCI‐HS serum that had been preincubated with heparin‐sepharose or carrier for 5 min on ice followed by centrifugation. TER was monitored for indicated time periods. Shown are representative data from three independent experiments.

Published studies suggest anti‐inflammatory and barrier protective properties of heparin in certain in vitro models (Nelson et al., 1993). Heparin was tested in this study for its capacity to inhibit HPAEC permeability caused by CCI‐HS sera. We found that CCI‐HS sera failed to induce significant TER decrease in the presence of heparin, and this barrier‐protective response was observed during the 15 h course of ECIS monitoring. It is noteworthy that heparin alone did not affect EC barrier properties (Figure 3a). To further verify the inhibitory activities of heparin on CCI‐HS‐induced EC permeability, CCI‐HS sera was first incubated with heparin‐sepharose and supernatants after precipitation of heparin‐sepharose beads by centrifugation were tested in permeability assays. The results indicated that the supernatants after serum incubation with heparin‐Sepharose completely lost their barrier disruptive activities (Figure 3C).

### 3.4 CCI‐HS sera induce the reduction of VE‐Cadherin expression in EC

**FIGURE 4:**
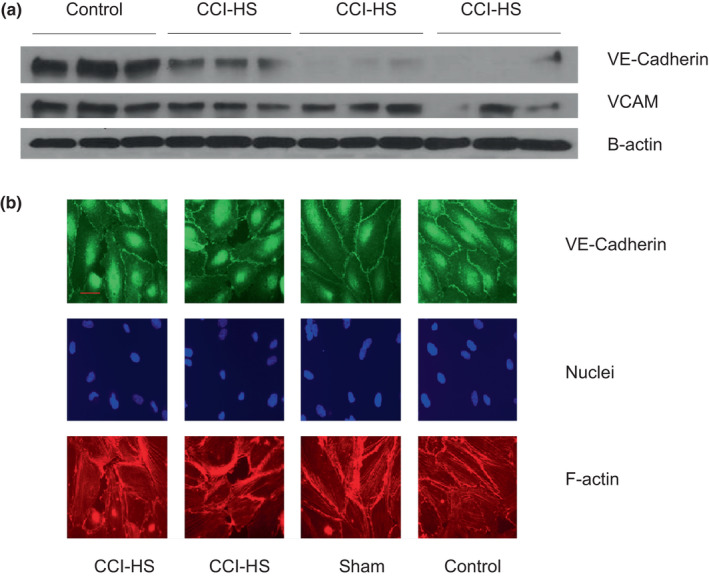
Effect of CCI‐HS serum on expression levels and intracellular localization of VE‐cadherin. Effects of CCI‐HS sera on EC junctional protein VE‐cadherin were assessed by WB analysis and immunofluorescence staining. (a) HPAEC were treated with serum from control or CCI‐HS rats (1:100, 3 h). VE‐cadherin expression was evaluated by western blot analysis of total cell lysates. (b) immunofluorescence staining of VE‐cadherin (top panels), nuclei labeling by DAPI (middle panels) and F‐Actin staining by Texas red phalloidin (bottom panels) of HPAEC monolayers incubated with sera from non‐operated, sham‐operated and CCI‐HS rats. Shown are representative images from four independent experiments. Bar: 20 μm.

To gain insight into the molecular mechanism whereby CCI‐HS serum caused disruption of endothelial barrier integrity, we analyzed the effects of CCI‐HS serum on the expression of VE‐Cadherin. HPAEC treated with CCI‐HS sera demonstrated significant reduction of VE‐Cadherin protein levels as shown by Western Blot analysis (Figure 4a). In support of this observation immunofluorescence staining of VE‐cadherin in EC treated with CCI‐HS serum also demonstrated reduction or break of continuity of VE‐Cadherin signal at cell junction area compared to those from the control cells or the cells treated with sham‐serum (Figure 4b).

## 4 DISCUSSION

The role of TBI as a factor leading to ARDS has been described in previous studies (Aisiku, Yamal, Doshi, Benoit, et al., 2016). It remains unclear which specific components in the TBI blood are responsible for the development of respiratory complications. Earlier studies indicated that levels of IL6, IL8, IL10 were elevated in the circulation of TBI patients and could have been associated with the development of ARDS (Aisiku, Yamal, Doshi, Benoit, et al., 2016). TBI‐induced ARDS was also associated with alteration in TIMP3 (Hendrickson et al., 2018), reduction of circulating microparticles (Midura et al., 2015), changes in APC (activated protein C) (Finigan, 2009) and HMGB1 (Braun et al., 2017). However, whether changes in serum composition induced by TBI directly affect EC barrier function remains unclear.

Our study shows that sera collected from the rats subjected to CCI and HS contain activities causing EC barrier dysfunction, while sera from the sham rats did not exhibit barrier disruptive effects. The CCI‐HS sera‐induced EC barrier dysfunction was rapid and reached the maximum within 30 min. Interestingly the barrier disruptive effects of TBI‐HS sera were deactivated by repeated freeze‐thaw cycles (data not shown) suggesting a protein nature of barrier‐disrupting substance(s). Similarly, freeze‐thawing causes inactivation of thrombin‐induced barrier‐disruptive activities. Dabigatran is a compound that directly targets thrombin and specifically inhibits the protease activities of thrombin (Hauel et al., 2002). ML161 is a Par1 antagonist in endothelial cells that block the signaling initiated by thrombin (Gandhi et al., 2018). The changes brought about by dabigatran and ML161 on CCI‐HS‐induced EC barrier dysfunction have provided strong evidence that thrombin is a component responsible for the development of EC barrier dysfunction during the early phase, but not in the later phase after TBI‐HS serum treatment.

TBI causes a disturbance in coagulation system leading to coagulopathy manifested as excessive or insufficient blood coagulation (McCully & Schreiber, 2013). Severe bleeding resulting from TBI leads to hemorrhagic shock (HS) and activation of coagulation cascades, which in turn impact on lung vascular integrity and permeability through aggregation of platelets, mobilization of fibrinogen, intravascular thrombosis, fibrinolysis, change of cytokine profiles, and other cellular and molecular events (Shaz et al., 2011). On the other hand, TBI without severe bleeding and hemorrhagic shock may still have strong effects on the balance of coagulation system (Tian et al., 2015). Tissue factor generated in brain, released into circulation and carried in microparticles following TBI activates thrombin‐initiated coagulation responses (Tian et al., 2015). Thrombin directly causes endothelial barrier dysfunction leading to vascular leak. An increase in plasma thrombin following traumatic brain injury and use of antithrombin as TBI therapeutics have been reported previously (Grenander et al., 2001). In a mouse TBI model, inhibition of thrombin by dabigatran was employed as an anti‐coagulation therapy to improve TBI outcome (Schaefer et al., 2014). Our studies further suggested that thrombin could be an important circulatory factor induced by CCI and HS that may have significant impact on endothelial cells and vascular integrity. Moreover, inhibition of thrombin activities may serve as a therapeutic modality in TBI patients with liability to develop ARDS.

Although the identity of the factors responsible for the late phase barrier disruptive activities remains unknown, it is evident that the late phase barrier disruptive activities were inhibited by heparin. Moreover, the activities could be removed from the serum by heparin‐sepharose, suggesting that the factor(s) responsible for the late‐phase barrier disruptive activities might be molecule(s) that directly bind to heparin. In addition to thrombin, there are other molecules reported to induce EC barrier dysfunction and at the same time, possess the capacity to interact with heparin. For example, histones are basic proteins that strongly bind to negatively charged heparin (Iba et al., 2015). Circulatory histones increase under certain pathological conditions, such as sepsis and trauma (Kutcher et al., 2012). These properties of histones as a factor contributing to TBI‐HS‐induced EC barrier dysfunction warrant further investigation.

Coagulopathy induced by traumatic brain injury may have profound impact on vascular integrity (Tian et al., 2015). Thrombin regulates platelet aggregation, coagulation, and EC barrier function as well (Birukov & Karki, 2018). It is unclear how CCI and HS lead to a surge of thrombin in circulation. Tissue factors generated during TBI and HS, released into circulation in the form of micro‐vesicle may trigger an increase of thrombin and thrombin activity in the blood or serum through coagulation factor Xa resulting in EC barrier dysfunction. However, this is clearly not a complete picture because a late phase barrier disruptive activity induced by the sera were not affected by inhibition of thrombin or thrombin receptor. Instead, the long‐lasting activity in late phase was blocked or removed by heparin and heparin Sepharose. Heparin is a well‐known anti‐coagulant and it binds to thrombin and many other proteins as well. Although serum factor(s) responsible for the barrier disruptive activity after thrombin remains unclear, it is very likely that the factor(s) could be a molecule or molecules directly interacting with heparin. Substantial evidence generated from both basic and clinical studies have pointed out a strong cause‐effect relationship between coagulopathy and ARDS. Loss of vascular integrity could be a consequence and at the same time an enhancing factor of coagulopathy. Based on the evidence that heparin could inhibit the barrier dysfunction induced by CCI‐HS serum, it raised the possibility that neutralization of humoral factors in blood by heparin in TBI patients could be a novel strategy to mitigate or reverse the lung injury.

VE‐Cadherin is one of the major junction proteins that maintain EC barrier integrity. The phosphoprotein is tightly controlled by signaling molecules including protein kinases and phosphatases. The EC barrier disruptive factors in CCI‐HS sera may function through one or more intracellular signaling cascades that lead to dissociation and break‐down of VE‐Cadherin proteins. Identification of the receptors activated by the serum factors as well as the down‐stream signaling cascades will help understanding the serum‐induced EC barrier dysfunction and a possible link with ARDS.

There are several limitations of this study. The serum samples used in this study were from rats euthanized only at 2 h following CCI plus HS. Effects of serum on EC barrier integrity at additional times following injury would provide additional insight into the mechanisms by which these samples disrupt EC barrier functions. In our earlier studies that used this polytrauma paradigm, the mortality ranged from approximately 20–60% (Proctor et al., 2015; Proctor et al., 2021). There was less than 20% mortality following CCI alone and no mortality with HS alone. The effects of CCI plus HS or single treatment (CCI or HS) on lung injury in vivo, as well as effects of serum obtained from animals exposed to CCI plus HS or CCI and HS alone on endothelial barrier dysfunction measured in vitro should be further quantified, dependent on the depth of cortical impact and the extent and duration of hemorrhagic shock. The histopathology performed on the lungs of rats used for testing serum on endothelial barrier function indicated severe lung inflammation, as reflected by myeloperoxidase immunohistochemistry. We did not, however, directly measure lung function in these animals. Future studies should include such assays along with serum effects on lung endothelial cells in rats and other species and with both males and females of different ages. Because HEX formulation was used in our study for resuscitation after HS, it is important to note that, although there are several reports suggesting both the beneficial and adverse effects of HEX on endothelial integrity or vascular permeability in different models (Dieterich et al., 2006; Guerci et al., 2019; Zausig et al., 2013), we have the evidence that hemorrhagic shock alone did not provoke severe condition to the animal as mentioned above. In addition, the results of current study suggest possible involvement of endogenously generated factors causing endothelial barrier disruption. Based on our present findings, it will be essential also to test for the ability of thrombin antagonists and heparin to protect against lung injury in vivo using several different animal models of TBI.

In summary, our study shows for the first time strong and sustained barrier disruptive effects of serum factors induced by TBI combined with hemorrhagic shock and demonstrates a key role of thrombin‐dependent mechanism in the acute phase of TBI‐HS‐induced vascular endothelial barrier dysfunction. Future studies are warranted to characterize the identity of other barrier‐disruptive molecule(s) with the ability to interact with heparin. The results suggest a potential therapeutic role of non‐anticoagulant modifications of heparin in mitigation of severe barrier‐disruptive effects of soluble factors appeared in the circulation of TBI patients and patients with severe polytrauma.

## AUTHOR CONTRIBUTIONS

Yunbo Ke, experimental design, experiments, data analysis and preparation of the manuscript; Julie L. Proctor: Animal model and sample collection, data analysis, writing and edit of the manuscript; Konstantin G. Birukov, Gary M. Fiskum, and Anna A. Birukova: Initiation of the collaborative study, supervision, experimental design, and edit of the manuscript; Thomas E. Grissom: Analysis and edit of the manuscript; Chenou Zhang, Juliana Medina, Catriona HT Miller, and Junghyun Kim, sample collection and experiments.

## FUNDING INFORMATION

The work was supported/partially supported by NIH fund: R01 HL146829 to KGB; US Air Force fund: FA8650‐15‐2‐6D21 to GF; and by UMB Department of Anesthesiology seed grant: 10023354 to YK/TG.

## CONFLICT OF INTEREST

There is no conflict of interest in this study.
